# Management of late-preterm and term infants with hyperbilirubinaemia in resource-constrained settings

**DOI:** 10.1186/s12887-015-0358-z

**Published:** 2015-04-12

**Authors:** Bolajoko O Olusanya, Tinuade A Ogunlesi, Praveen Kumar, Nem-Yun Boo, Iman F Iskander, Maria Fernanda B de Almeida, Yvonne E Vaucher, Tina M Slusher

**Affiliations:** Centre for Healthy Start Initiative, 286A, Corporation Drive, Dolphin Estate, Ikoyi, Lagos Nigeria; Department of Paediatrics, Olabisi Onabanjo University Teaching Hospital, Sagamu, Nigeria; Department of Paediatrics, Neonatal Unit, Postgraduate Institute of Medical Education and Research, Chandigarh, India; Department of Population Medicine, Faculty of Medicine and Health Sciences, Universiti Tunku Abdul Rahman, Bandar Sungai Long, Selangor, Malaysia; Department of Paediatrics, Cairo University, Cairo, Egypt; Division of Neonatology, Federal University of São Paulo-UNIFESP, São Paulo, SP Brazil; Division of Neonatal/Perinatal Medicine, School of Medicine, University of California at San Diego, San Diego, USA; Division of Global Paediatrics, University of Minnesota, Minneapolis, Minnesota USA; Hennepin County Medical Centre, Minneapolis, Minnesota USA

**Keywords:** Clinical guidelines, Developing countries, Exchange transfusion, Hyperbilirubinaemia, Kernicterus, Neonatal jaundice, Newborn care, Phototherapy

## Abstract

Hyperbilirubinaemia is a ubiquitous transitional morbidity in the vast majority of newborns and a leading cause of hospitalisation in the first week of life worldwide. While timely and effective phototherapy and exchange transfusion are well proven treatments for severe neonatal hyperbilirubinaemia, inappropriate or ineffective treatment of hyperbilirubinaemia, at secondary and tertiary hospitals, still prevails in many poorly-resourced countries accounting for a disproportionately high burden of bilirubin-induced mortality and long-term morbidity. As part of the efforts to curtail the widely reported risks of frequent but avoidable bilirubin-induced neurologic dysfunction (acute bilirubin encephalopathy (ABE) and kernicterus) in low and middle-income countries (LMICs) with significant resource constraints, this article presents a practical framework for the management of late-preterm and term infants (≥35 weeks of gestation) with clinically significant hyperbilirubinaemia in these countries particularly where local practice guidelines are lacking. Standard and validated protocols were followed in adapting available evidence-based national guidelines on the management of hyperbilirubinaemia through a collaboration among clinicians and experts on newborn jaundice from different world regions. Tasks and resources required for the comprehensive management of infants with or at risk of severe hyperbilirubinaemia at all levels of healthcare delivery are proposed, covering primary prevention, early detection, diagnosis, monitoring, treatment, and follow-up. Additionally, actionable treatment or referral levels for phototherapy and exchange transfusion are proposed within the context of several confounding factors such as widespread exclusive breastfeeding, infections, blood group incompatibilities and G6PD deficiency, which place infants at high risk of severe hyperbilirubinaemia and bilirubin-induced neurologic dysfunction in LMICs, as well as the limited facilities for clinical investigations and inconsistent functionality of available phototherapy devices. The need to adjust these levels as appropriate depending on the available facilities in each clinical setting and the risk profile of the infant is emphasised with a view to avoiding over-treatment or under-treatment. These recommendations should serve as a valuable reference material for health workers, guide the development of contextually-relevant national guidelines in each LMIC, as well as facilitate effective advocacy and mobilisation of requisite resources for the optimal care of infants with hyperbilirubinaemia at all levels.

**Electronic supplementary material:**

The online version of this article (doi:10.1186/s12887-015-0358-z) contains supplementary material, which is available to authorized users.

## Introduction

Neonatal hyperbilirubinaemia is a leading cause of hospital admission/re-hospitalisation in the first week of life globally [1-3]. Timely and appropriate treatment with phototherapy and/or exchange transfusion are effective in controlling excessive bilirubin levels in the affected infants [4,5]. Otherwise, severe hyperbilirubinaemia may progress to acute bilirubin encephalopathy (ABE) or kernicterus with a significant risk of mortality in newborns [6-8]. Survivors may also acquire long-term neurodevelopmental sequelae such as cerebral palsy, sensorineural hearing loss, intellectual difficulties or gross developmental delays [9-13]. It is estimated that, worldwide, severe hyperbilirubinaemia affects at least 481,000 term or near-term newborn babies annually, of whom 114,000 die and more than 63,000 survive with moderate or severe disability [14,15]. At least, 75% of the affected infants reside in sub-Saharan Africa and South Asia [14].

In low- and middle-income countries (LMICs), delay in seeking care for infants with hyperbilirubinaemia as well as delay in providing appropriate treatment when affected infants present in health facilities is commonly reported [16,17]. Where phototherapy devices are available, if at all, lack of relevant guidelines or inadequate knowledge of essential requirements for effective treatment results in frequent and potentially avoidable exchange transfusions [18-21]. We, therefore, set out to identify key considerations for the effective management of late-preterm and term infants (≥35 weeks of gestation) with significant hyperbilirubinaemia presenting at health facilities in LMICs.

## Review

### Methodology

Guidelines for the management of hyperbilirubinaemia in high-income countries are unlikely to address the peculiar challenges in LMICs without appropriate modification [17,22,23]. Adaptation of existing evidence-based guidelines from one geographical, economic and socio-cultural context to another is an internationally accepted alternative to the more costly, time-consuming, *de novo* guideline development for improved health care delivery [24]. In this report, we followed relevant protocols of the WHO Handbook for Guideline Development [25], ADAPTE Guideline Adaptation Toolkit [26], and AGREE II-Global Rating Scale [27], for the adaptation of clinical practice guidelines. Based on prior in-depth reviews of the literature from 1970 to 2013 on the burden of neonatal hyperbilirubinaemia and current management practices in LMICs [17,22], we identified four major themes for improving the care of affected infants namely: primary prevention, early detection and monitoring, treatment and follow-up. We then undertook a review of existing guidelines (see Additional file 1: Table S1) and relevant literature from both high-income and LMICs to identify key issues relevant to improved care at primary, secondary and tertiary levels in LMICs [28,29]. The existing guidelines were rated individually by the core working group (CWG) and one external content methodologist who had no prior involvement with generating these guidelines. The overall average score for each guideline was computed based on the seven components of the AGREE-II instrument: methodology, presentation, completeness, appropriateness, overall quality, disposition for personal use and likelihood of recommending the guideline to others [27]. We developed a practice framework for different levels of newborn care based on essential tools and skills considered appropriate for each level of care. The proposals aimed at balancing the safe, effective, patient-centred, timely, efficient and equitable components of quality care enunciated by the Institute of Medicine [30], as well as minimising the risk of unintended harms such as costly, unnecessary overtreatment or increased parental anxiety. Where scientific evidence was lacking or limited, proposed actions were based on consensus among the CWG using the Delphi process [31]. The draft and final proposals were critically reviewed by an international panel of experts for scientific soundness and practicality. The experts were identified and agreed by the CWG based on their independently verifiable work on the subject-matter and with a view to achieving a fair representation from all world regions. While the expert panel review was not intended as individual endorsement of the entire framework, all comments and queries were carefully addressed by the CWG in subsequent revisions. Authors made reasoned judgment where contradictory views were expressed by panel members on an issue [25-27]. For clarity and consistency the key terminologies and definitions used in this report are summarised in Table 1 (also Additional file 2).

**Table 1:** **Terminologies and definitions adopted in this paper**

**Terminology**	**Definitions**
**Clinically significant hyperbilirubinaemia**	*Significant hyperbilirubinaemia:* any unconjugated bilirubin level requiring treatment with phototherapy which varies with post-natal age and aetiology (typically TSB ≥12 mg/dL (205 μmol/L) in many LMICs).
	*Severe hyperbilirubinaemia*: Bilirubin levels at/near exchange transfusion levels based on post-natal age and aetiology (typically TSB ≥20 mg/dL or 342 μmol/L in many LMICs) and/or any elevated TSB associated with signs of acute bilirubin encephalopathy.
	*Bilirubin encephalopathy*: abnormal neurological signs and symptoms caused by bilirubin toxicity to the basal ganglia and various brainstem nuclei.
	*Acute bilirubin encephalopathy (ABE)*: acute manifestations of bilirubin toxicity seen within fourteen days after birth. Classic early signs include poor feeding, lethargy and tone abnormalities progressing to high-pitched cry, increasing hypertonia - especially of extensor muscles, with retrocollis, opisthotonus and obtundation in association with the kernicteric facies.
	*Kernicterus*: Permanent or chronic neurologic damage, including choreo-athetoid cerebral palsy, enamel dysplasia, paralysis of upward gaze, hearing impairments including auditory neuropathy spectrum disorders.
**Low- and middle-income countries (LMICs)**	The target population for this review consists of the 91 countries with per capita Gross National Income (GNI) of ≤ US$6,000 using the Human Development Report 2013 by the United Nations Development Program (UNDP) as there is no single definition of “resource-poor countries” in the literature and developmental status varies greatly among the approximately 140 countries classified as LMICs by the World Bank [17]. (see Additional file 2: Table S2)
**Levels of health care delivery**	Three levels of healthcare delivery were considered: primary, secondary and tertiary. Typically, the primary level consists of community health centres and outposts managed by community health workers. Secondary/first-level referral centres include district or general hospitals while the tertiary level consists of specialist or teaching hospitals.

### Levels of intervention and required facilities for severe jaundice

The average AGREE II-GRS ratings of the 21 guidelines reviewed ranged from 41% for Ghana and 99% for UK’s NICE (see Additional file 1: Table S1). Priority was given to guidelines with high quality scores (≥70%) except for issues pertinent to clinical practice in LMICs but not explicitly addressed by these guidelines such as factors accounting for delays in seeking and receiving appropriate care [17]. Only four guidelines (Ghana, India, Kenya and WHO) were from eligible LMICs, and two (Ghana and WHO) did not meet the 70% quality rating threshold. The American Academy of Paediatrics (AAP) guideline was the benchmark for the majority of high scoring national guidelines including NICE [4,5]. The interventions and tools proposed for each level of health care delivery are summarised in Table 2.

**Table 2:** **Levels of intervention and suggested tools for managing neonatal hyperbilirubinaemia in low and middle-income countries**

Proposed tasks or tests [Applicable level of care]*	**Suggested tools and facilities**
**Primary prevention**	
• Education of existing and expectant mothers, families and health care providers on [P, S & T]:	• Educational materials including posters and audio-visual aids where available, pictures and/or video clips of infant survivors of BIND and/or Kernicterus. This material should include signs of both early and late ABE/BIND and potential long-term consequences of ABE/BIND for both the community and the health care providers. [P, S & T]
o The transient physiologic course but with potential to increase to harmful levels and it's variability from baby to baby	
o The avoidance of haemolytic substances (including camphor/naphthalene balls, menthol-containing powder, creams and balms, e.g. Wintergreen oil).	
o The benefits of early detection accompanied by timely and appropriate treatment in health facilities adequately-equipped for newborn care.	Access to laboratory appropriately resourced for clinical investigations. [S & T]
o Discouraging traditional therapies as well as indiscriminate use of self-prescribed medications e.g. ampicillin-cloxacillin.	
o Recognition of acute bilirubin encephalopathy/Bilirubin-Induced Neurologic Dysfunction (BIND)	
o The value of “clean birth” to prevent or minimize the risk of infection (sepsis)	
• Referral to secondary or tertiary centers of all preterm babies (<35 weeks gestation) and surveillance for full-term infants with history of medically-treated jaundice in a sibling presenting at primary health centers. [P]	
• Promotion and support for successful breastfeeding. [P, S & T]	
• Screening of expectant mothers for the risk of blood group incompatibilities using routine ABO & Rh with counseling on the importance of Rh immunoglobulin ensuring availability when indicated. [P, S & T]	
• Judicious use of oxytocin during labor. [S & T]	
• Identification of babies with extensive bruises, cephalhaematomas and at risk for concealed haematomas e.g. those from difficult deliveries. [P, S & T]	
• Request blood test to rule out Glucose-6-phosphate dehydrogenase (G6PD) deficiency in high-risk populations. [S & T]	
• Early phototherapy for infants with hemolytic diseases. [S & T]	
**Early detection, diagnosis and monitoring**	
• Routine examination of all newborns within 24 hours of birth and the next 48 hours for possible jaundice. [P, S & T]	• Well-lit examination room or nursery with natural daylight (minimum). [P, S & T]
• If jaundice is suspected, examine infant naked in a well-lit room or, preferably in natural daylight near a window guided by Kramer’s chart (Additional file 3: Figure S1). Recognize that estimation of the degree of hyperbilirubinaemia based on visual signs of jaundice can lead to errors, particularly in darkly pigmented infants. Blanching of the gum may be more reliable and helpful in dark skinned babies. [P, S & T]	• Transcutaneous (TcB) Bilirubinometer e.g. JM103® or Bilicheck® (minimum) or Icterometer. [P, S & T]
	• Rapid micro device for total plasma/serum bilirubin (TSB) (minimum). [S & T]
	• Bilirubinometer for total serum & direct bilirubin measurement (minimum). [S & T]
• If jaundice is visible, measure the total serum bilirubin (TSB) or transcutaneous bilirubin (TcB) level. TcB values above 12 mg/dl (205 μmol/L) should be cross-checked where possible with TSB measurement. [P, S & T]	• Access to laboratory facilities for: [S & T]
	o Blood group, Rh, G6PD tests (minimum)
	o Components of sepsis screen such as Complete Blood Count, Blood/Urine/CSF cultures, CRP and/or other rapid screening tests
• Establish if infant has early signs of acute bilirubin encephalopathy (ABE) or qualifies as high risk including possible hemolytic diseases, hypothermia, hypoglycemia, or sepsis (see Algorithm in Figure 1). [P, S & T]	o Metabolic screening e.g. hypothyroidism, galactosaemia when indicated.
• Follow the Algorithm and Table 3 for actionable levels. [P, S & T]	
• Ensure follow-up of infants discharged before 48 hours after delivery especially those with established risk factors within 1–2 days of discharge (take advantage of BCG visit and any other times infants <2 weeks are seen). [P, S & T]	
**Treatment**	
• Follow the Algorithm and Table 3 for actionable levels after country specific adaptations. [S & T]	• Effective Phototherapy Units [S & T]
• When indicated (particularly, in the presence of isoimmune haemolytic diseases), ensure early treatment of newborns with intensive phototherapy to minimize the need for exchange transfusion. [S & T]	o Special Blue-light Phototherapy Unit (ideal)
	o Light emitting diodes (LED) Phototherapy Unit (ideal)
• Ensure that the irradiance levels of phototherapy units are periodically monitored and the recommended specifications strictly followed. [S & T]	
	o Fluorescent white or blue bulbs (minimum)
• Be familiar with simple and inexpensive adjustments that can significantly improve the effectiveness of phototherapy devices. [S & T]	• Irradiance Meters (spectro-radiometers) (ideal). [S & T]
	• Access to blood bank with fresh (ideally less than 3 days old) whole blood for ET appropriately compatible with mother and baby (O and Rh specific). [S & T]
• Maximize irradiance by placing the units as close as possible without overheating the infants (usually 10–20 cm above the baby if using cool lights (unless specified otherwise by manufacturer), using reflecting materials on all sides of cots, exposing as much of the baby as possible (thereby maximizing spectral power [irradiance x size of irradiated area]); and change florescent tubes according to manufacturer’s recommendations if available or periodically (8–12 weeks) if unable to measure irradiance levels [S & T]	
	• Intravenous immunoglobulins (IVIG) may be useful when nearing ET but existing evidence is not conclusive. [T]
	• Laboratory facilities for albumin [S & T]
• Ensure that the eyes are covered but keep the cover small to maximize surface available for PT. [P, S & T]	o Consider bilirubin-albumin ratio in addition to but not in lieu of total bilirubin level as a factor in determining the need for an exchange transfusion.
• Ensure that male genitals are covered (controversial) unless for infants nearing exchange transfusion level. [P, S & T]	
• Ensure that babies are placed in cots not incubators when under phototherapy unless they are hypothermic. [S & T]	
• Ensure that blood samples for relevant investigations are collected and refrigerated before initiating exchange transfusion and any blood samples are protected from light including the PT light. [S & T]	• If available, consider the use of duly approved filtered sunlight phototherapy (FS-PT) in tropical settings with irregular electricity supply and lack of adequate or functional conventional phototherapy (CPT) units with careful and frequent monitoring of infants for temperature fluctuations. [ P & S]
• Ensure that an infant with clinical signs of moderate-severe ABE receives exchange transfusion promptly. Place the infant under the best phototherapy available while preparing for the exchange transfusion.[S & T]	
• Ensure that the infant remains adequately hydrated and is breastfeeding well/feeding well. [P, S & T]	
• Avoid drugs that compete for albumin binding such as sulfonamides, ceftriaxone, and acetylsalicylic acid. [S & T]	
**Follow-up evaluation**	
• Assessment of non-jaundiced infants on days 3 and 5. [P, S & T]	• Tools for age-appropriate developmental assessment. [S & T]
• Assessment of jaundiced infants regularly in the first 7–10 days or until jaundice is clearly resolved. [P, S & T]	
	• Automated Otoacoustic Emissions (AOAE). [S & T]
• Educate parents on the need for a follow-up neuro-developmental assessment of all infants treated for severe hyperbilirubinaemia with intensive phototherapy or exchange transfusion or with a history of such treatment at age 3–6 months. [P, S & T]	• Automated Auditory Brainstem Response (AABR). [S & T]
	• Access to diagnostic evaluation with Auditory Brainstem Response (ABR) (ideal). [T]
• Ensure that, at the minimum, such developmental assessment includes auditory brainstem response audiometry, language processing/language development and clinical evaluation of abnormalities of tone, posture and movements for infants with signs of ABE/BIND, who had exchange transfusion and those with a bilirubin level of >20 mg/dL. [S & T]	• Consider Magnetic Resonance Imaging (MRI) for early detection of potential neurotoxicity if readily available, can be done without sedation and does not delay treatment. [T]
• Disseminate information on the local providers of age-appropriate developmental evaluation of infants and young children to the affected parents on discharge or during any subsequent clinical consultations. [S & T]	

*Level of care where task/test should be available routinely: Primary/Community Health Center [**P**], Secondary/District Hospital [**S**], Tertiary/Children’s’ Hospital [**T**]. For a comprehensive list of essential infrastructural and human resources typically required for secondary/district hospitals see: UNICEF India. Toolkit for Setting up of Special Care Units, Stabilization Units and Newborn Care Units. New Delhi: UNICEF India, 2009.

➢ → *Conventional Phototherapy (CPT)*: Phototherapy in which intensity of blue light (400–520 nm) with a peak wavelength of 450 ± 20 nm not less than 8 μW/cm^2^/nm is applied to the greatest possible surface area of the infant. The light sources are usually special blue fluorescent lamps, compact florescent lamps (CFL) or halogen spotlights. If none of these are available, ordinary commercial white/daylight fluorescent lights should be considered, but brought as close as possible (10-20 cm) to the baby without overheating.

➢ → *Light-emitting diode Phototherapy (LED-PT)*: Phototherapy devices which emit most of their light in the 450–470 nm spectrum. This range corresponds to the peak absorption wavelength (458 nm) at which bilirubin is broken down. Blue LEDs are power efficient, portable devices with low heat production so that they can be placed very close to the skin of the infants without any apparent untoward effects.

➢ → *Intensive Phototherapy (IPT)*: Phototherapy in which a high intensity of blue light (400–520 nm) ≥30 μW/cm2/nm is applied to the greatest possible surface area of the infant. In usual clinical situations, this will require special high-intensity fluorescent tubes, or CPT lamps placed approximately 30 cm (10 cm for cool blue light) above the infant, who can be nursed in a bassinet.

➢ → *Filtered Sunlight Phototherapy (FS-PT)*: Treatment with specially filtered sunlight using custom pre-tested window-tinting films that protect against potentially harmful ultra-violet and infra-red rays.

### Primary prevention

The contribution of maternal/family knowledge gaps regarding the importance of neonatal jaundice commonly manifesting in late presentation of infants with severe hyperbilirubinaemia to health services in LMICs is well documented [17]. Mothers and families are able to detect jaundice from yellowish discolouration of the skin in their newborns accurately, if appropriately educated [4]. Educating pregnant women, especially primigravidae during antenatal clinics, on the risks and adverse consequences of severe hyperbilirubinaemia, avoidance of potentially harmful traditional/herbal therapies and the mis(use) of haemolytic agents should be a priority [17]. Routine determination of mother’s blood type and timely provision of anti-D globulin should be widely promoted to prevent Rh and neonatal jaundice due to other haemolytic diseases [14,32]. Educational interactions with mothers and families must also recognise and seek to address common barriers to appropriate health-seeking behaviour for childhood illnesses [17]. Although most infants are born outside hospitals in many LMICs, pre-discharge counselling of mothers who deliver in hospitals on the risks of hyperbilirubinaemia after discharge should be considered.

Infants that are exclusively breastfed have an increased risk for severe hyperbilirubinaemia in the first 2 to 5 days of life compared to formula-fed infants [33]. It is therefore, essential to provide good lactation support to all mothers at all levels of care to increase successful breastfeeding, at least 8–12 times a day, as breast-milk benefits outweigh the risk [4,5,33]. Mothers, families and their jaundiced infants will also be best served by information provided during antenatal care, about hospitals in their communities that are able to provide requisite support for neonatal hyperbilirubinaemia [17].

Mothers who deliver at home, especially those who do not attend antenatal clinics present a special challenge that must be appropriately addressed in various communities. The inclusion of neonatal jaundice in the WHO recommended training on essential newborn care for traditional/home birth attendants, community and lay health workers should be considered in such settings [34-36]. The training should also be geared towards avoidance of haemolytic agents or traditional therapies, early recognition of the onset of jaundice by mothers and care givers, and surveillance for timely presentation to the nearest health facility [17].

### Early detection, diagnosis and monitoring

Early identification of infants at risk of severe hyperbilirubinaemia is an essential component of newborn care. All newborns at all levels should be examined within 24 hours of birth and in the following two days. Mothers and other care-givers should be encouraged to look for jaundice by blanching the skin (on the nose in particular), looking at the gums and examining the eyes [4]. The use of Kramer’s chart (see Additional file 3: Figure S1) especially in primary care settings remains valuable despite its limitation in correlating with the severity of jaundice [37,38]. So also is blanching of the gums possibly with an icterometer, particularly in dark-skinned babies [39]. Healthcare professionals and parents are capable of recognizing jaundice, but not very good at assessing its severity [4]. Notwithstanding, this visual assessment is generally more reliable and helpful in ruling out hyperbilirubinaemia than estimating bilirubin levels [4].

**Figure 1:**
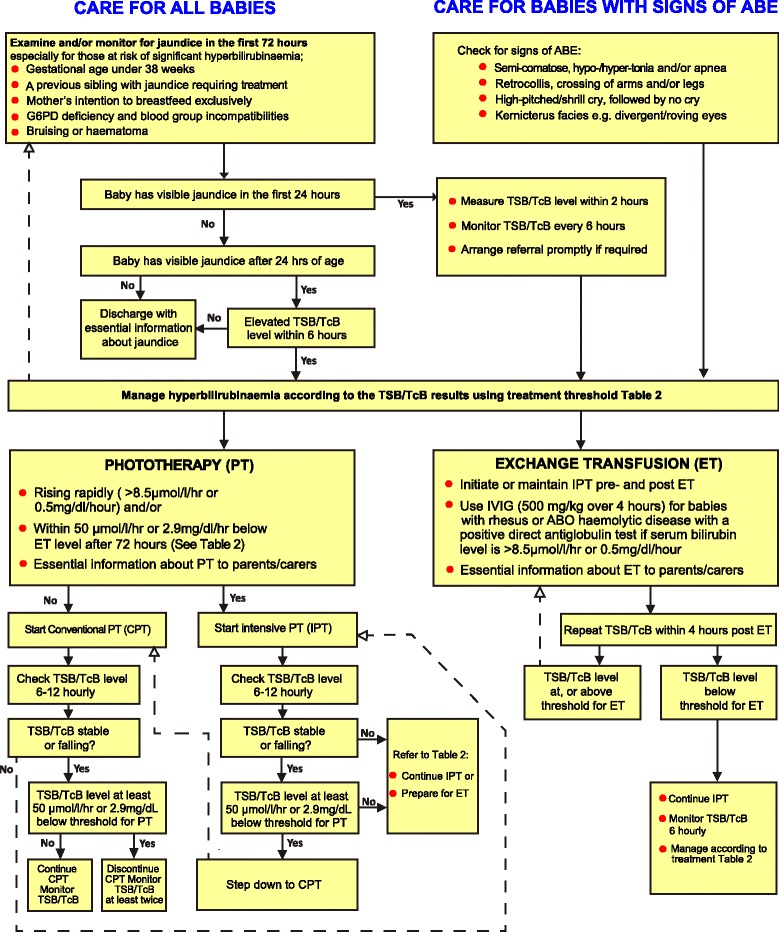
Algorithm for the care of newborns with hyperbilirubinaemia in LMICs.

The suggested pathways of care for all babies, adapted from NICE guidelines, are described in Figure 1 [4]. At a minimum, infants with gestational age <38 weeks, from high-risk racial groups, previous sibling(s) with a history of treated jaundice, visible jaundice in the first 24 hours of birth, family history of G6PD deficiency or blood group incompatibilities, should be considered as high risk and should be monitored for hyperbilirubinaemia at all levels of care [4,5]. Ongoing training of health workers on the signs and symptoms of ABE including the use of a protocol for bilirubin-induced neurologic dysfunction (BIND) is essential for facilitating timely referral and intervention (see Additional file 4: Table S3) [40,41]. Subtle, moderate and occasionally even advanced BIND is likely reversible [42,43]. Timely detection and treatment of BIND as suggested by the AAP and others is therefore, useful in forestalling the progression of this potentially devastating condition.

The objective tests for estimating and monitoring the degree of jaundice are transcutaneous bilirubin (TcB) and/or TSB. TcB is a non-invasive, portable screening tool ideally used to determine the need for the more accurate TSB which requires a venous or capillary blood sample [44-47]. TcB values above 12 mg/dl (205 μmol/L) should be checked where possible with TSB measurement [47]. TcB becomes unreliable after commencement of phototherapy unless measurements are taken from an area of skin that has been shielded from phototherapy with a photo-opaque patch [47]. However, currently these devices, especially TSB, may not be readily affordable in many resource-limited settings. Low-cost and minimally invasive point-of-care tools for plasma/serum bilirubin measurements are currently being piloted and hold promise for LMICs [48]. The interpretation of TSB/TcB and the recommended actions are provided in Table 3 [47-51]. Besides TSB estimation, access to laboratory facilities for real-time clinical investigations should at a minimum include evaluation of blood group incompatibilities and G6PD status [4,5].

**Table 3:** **Suggested actionable treatment or referral TcB and/or TSB (mg/dL or μmol/L) levels in infants with hyperbilirubinaemia**

**Age (in hours)**	***Repeat TcB/TSB daily if baby is not under PT***	***Begin CPT or Refer at TcB/TSB***	***Begin IPT or Refer at TcB/TSB***	***Frequency of Monitoring TcB or TSB under PT***	***Consider ET at TcB/TSB***
0-12	any visible jaundice	2-3 (34–51)	>3 (51))	6 hrly	10-12 (171–205)
>12-24	≥4 (68)	4-5 (68–86)	>5 (86)	6 hrly	11-13 (188–222)
>24-36	≥5 (86)	6-7 (103–120)	>7 (120)	6 - 24 hrs	13-15 (222–257)
>36-48	≥7 (120)	7-9 (120–154)	>9 (154)	6 - 24 hrs	14-16 (239–274)
>48-60	≥9 (154)	9-11 (154–188)	>11 (188)	6 - 24 hrs	15-17 (257–291)
>60-72	≥10 (171)	10-12 (171–205)	>12 (205)	6 - 24 hrs	16-18 (274–308)
>72-84	≥10.5 (180)	11-13 (188–222)	>13 (222)	6 - 24 hrs	16-19 (274–325)
>84-96	≥11 (188)	12-14 (205–239)	>14 (239)	6 - 24 hrs	17-20 (291–342)
>96-108	≥11.5 (197)	12-15 (205–257)	>15 (257)	6 - 24 hrs	17-20 (291–351)
>108	≥12 (205)	13-16 (222–274)	>16 (274)	6 - 24 hrs	17-20 (291–351)

TcB (Transcutaneous bilirubinometry), TSB (Total serum bilirubin), PT (Phototherapy), CPT (Conventional PT) ≥10 μW/cm^2^/nm, IPT (Intensive PT) ≥30 μW/cm^2^/nm, ET (Exchange transfusion).

AAP (American Academy of Pediatrics), LMICs (Low and middle-income countries), G6PD (Glucose-6-Phosphate Dehydrogenase).

Notes:

• → The above levels are primarily adapted from the high/medium risk categories of AAP guidelines. Generally, levels of 2 mg/dL (34 μmol/L) below AAP recommendations are proposed due to multiple confounding factors such as the high risk status of many infants in LMICs, the limited facilities for clinical investigations, quality variability of phototherapy devices and the high incidence of ABE/kernicterus in many LMICs [e.g. see Guidelines #15 & 17 in Additional file 1: Table S1]. Phototherapy and especially exchange transfusion levels at or near those recommended by the AAP or NICE exchange guidelines should be strongly considered in tertiary care settings with intensive phototherapy.

• → These proposals may be adjusted as appropriate depending on the available facilities in each clinical setting and the risk profile of the infant with a view to avoiding overtreatment or under-treatment.

• → Factors that place infants at higher risk in many LMICs include but are not limited to widespread exclusive breastfeeding, G6PD deficiency, unrecognised haemolysis such as blood group incompatibilities and sepsis/infection.[e.g. see *Olusanya BO, Osibanjo FB, Slusher TM: Risk factors for severe hyperbilirubinaemia in low and middle-income countries: a systematic review and meta-analysis. PLoS ONE 2015,10(2):e0117229*.]

• → The distinction between when to begin CPT versus IPT is important in LMICs due to the sub-optimal quality of phototherapy and the limited number of IPT units in many settings. No such clear distinction exists in the AAP guidelines.

• → If TcB level indicates PT, verify level using TSB measurement if available. It is acceptable to determine need for TSB with a TcB and it may be acceptable to use TcB alone (under a photo-opaque patch) to follow infants under CPT. TcB values above 12 mg/dl (205 μmol/L) should be cross-checked where possible with TSB measurement.

• → All blood specimens for TSB measurement must be shielded from light to prevent photo-degradation of the sample serum bilirubin.

A centre or hospital, at any level, not appropriately resourced to provide the required treatment should refer promptly to the closest suitable health facility.

For infants delivered in hospitals and discharged before 48 hours, follow up assessment within 1–2 days after discharge with a TcB, or at a minimum, physical examination should be considered. Table 3 may be used to assess the risk of subsequent hyperbilirubinaemia at the time of discharge. Infants who present in the first week of life should be routinely examined for possible jaundice.

### Treatment

Phototherapy and exchange transfusion are well-established as the most effective treatments for severe hyperbilirubinaemia [4,5]. The proposed actionable TSB/TcB levels for phototherapy and exchange transfusion reflecting available evidence on current practices for the care of jaundiced infants in LMICs are presented in Table 3 [17,22,52]. We adopted the tabular format in the Kenya 2013 guidelines (see Additional file 1: Table S1) for ease of reference at all levels of care. These conservative bilirubin levels may be warranted in settings where the incidence of severe hyperbilirubinaemia is high, late presentation common, determination of haemolytic risk (Blood type/Rh/G6PD) is not possible routinely at birth, and quality of phototherapy is sub-optimal. Higher levels for phototherapy and especially exchange transfusions at or near those recommended by the AAP or NICE guidelines should be strongly considered in tertiary care settings with facilities for intensive phototherapy. A centre or hospital at any level that is not appropriately resourced to provide the required treatment should promptly refer the infant to the closest, appropriate health facility.

There are various phototherapy devices using different light sources: fluorescent tubes, halogen lamps and light emitting diodes (LED). An effective phototherapy device should produce specific blue-light wavelengths (peak emission: 450 ± 20 nm), preferably in a narrow bandwidth to about 80% of an infant’s body surface area [18]. Conventional phototherapy (CPT) should have an irradiance of at least 8-10 μW/cm^2^/nm and intensive phototherapy should have an irradiance of ≥30 μW/cm^2^/nm (from either a single or multiple phototherapy units). LED devices are as effective as other light sources in decreasing TSB but have special advantages in LMICs [53,54]. They are more power efficient, portable, weigh less, have a longer life span and lower heat production, making them more suitable for intensive phototherapy than fluorescent bulbs. Irradiance meters for monitoring PT units should be readily available, as well as spare replacement bulbs.

Costs of providing intensive or special care for jaundiced newborns could be prohibitive, next only to that of caring for preterm babies in LMICs [55]. However, the development of affordable phototherapy devices and using simple inexpensive enhancements such as hanging white reflecting material around cots (being careful to avoid overheating particularly from halogen lamps), changing bulbs regularly and reducing the distance between baby and lamps, improve the effectiveness of phototherapy [18,19,56,57]. WHO maintains a valuable compendium of innovative and low-cost technologies including phototherapy devices recommended for LMICs [58]. Whatever the light-source, the effectiveness of phototherapy devices can be compromised by erratic power supply, inadequate skin exposure from overcrowding with multiple infants placed under a single device, sub-optimal irradiance levels, and poor device maintenance [18,19]. Failure to address these issues is likely to increase the frequency of avoidable exchange transfusion. It is, therefore, essential that phototherapy devices are properly monitored, regularly maintained, and staff well trained on how to care for infants receiving phototherapy. If enough effective phototherapy devices are not available in very busy resource-constrained settings, clinical judgement as to which infants should receive priority based on the risk of ABE may be required. Under such conditions, a BIND assessment can aid decisions for intervention [40,59].

Guidelines for hyperbilirubinaemia in many high-income countries prohibit exposure to sunlight as a form of treatment [4,5,60]. This is primarily due to safety concerns regarding potentially harmful infrared and ultraviolet rays and possible sunburn. In Ghana, exposure of jaundiced infants to sunlight is acknowledged as a treatment option [61], but WHO guidelines and some other developing countries like Malaysia, India and Kenya discourage or have not made provisions for sunlight therapy (see Additional file 1: Table S1). However, mothers and caregivers with or without the support of health workers continue to expose their jaundiced babies to direct sunlight even in high-income countries [62-66]. Emerging evidence suggests that the potential risks can be mitigated through specially filtered film canopies which have been successfully piloted in West Africa [67,68]. However, their use is still experimental and limited to daytime care at periods with favourable climatic conditions. In remote tropical locations where access to conventional treatment is not assured, the trade-off between the risk of ABE/kernicterus and of exposure to sunlight remains a challenge that requires individual and informed judgment call in the patient’s best interest.

Immediate exchange transfusion is warranted when phototherapy has failed to effectively curtail the rate of bilirubin rise and the TSB/TcB measurement is near exchange levels or the infant has any of the signs of moderate to advanced ABE regardless of the TSB/TcB levels (Figure 1, Table 3). This treatment is most likely to be available at tertiary hospitals with trained personnel and facilities for special care, including monitoring and resuscitation capabilities. As exchange transfusion is not without risks [69-71], its frequency should be minimised as far as practicable. Exchange transfusion with G6PD deficient donor blood should be avoided where possible as this may prolong time under phototherapy and result in repeat exchange transfusions [72]. Similarly, blood should be screened for HIV and hepatitis. Rh-negative blood should be used for neonates with Rh-isoimmunisation while O group should be used for neonates with ABO incompatibility.

The evidence in support of pharmacotherapies such as D-penicillamine, phenobarbital, metalloporphyrins, clofibrate, bile salts, laxatives and bilirubin oxidase are inconclusive and these interventions have not been recommended [73]. Likewise traditional herbs or medications used to treat newborn jaundice in many home settings are not recommended.

### Follow-up evaluation

The manifestations of BIND such as cerebral palsy, auditory impairments, epilepsy, gross motor deficits, behavioural problems and intellectual difficulties are not uncommon in LMICs [9-13]. Follow-up evaluation of survivors of severe hyperbilirubinaemia for potential neurodevelopmental sequelae is necessary to facilitate early detection and intervention for the affected infants. This must be considered as an integral part of any clinical protocol for the management of infants who have been treated for severe hyperbilirubinaemia. Because hearing impairment, including auditory neuropathy spectrum disorders in the first year of life is often not clinically apparent, at-risk infants must be objectively evaluated within the first three months and monitored for language development in the first two years of life irrespective of the hearing test result. Referral to the audiology or otolaryngology unit of a tertiary hospital should therefore be considered. Several low-cost and simple-to-use validated tools for early developmental assessment as well as approaches to effective intervention in resource-limited settings have also been documented [74,75].

### Limitations

While we set out to ensure that our recommendations are realistic and consistent with the prevailing conditions in most LMICs, the methodological approach used in this document deserves clarification. We followed standard protocols for guideline adaptation including a comprehensive review of the priority issues to be considered, the formation of a working group, quality rating of existing guidelines and independent review by a panel of leading clinicians and experts on newborn care from different world regions [25-27]. However, we did not grade specific studies in support of various proposals in this paper, more so because the body of evidence was predominantly adapted from existing guidelines or supported by the best available data from LMICs [17,22,76]. Neonatal hyperbilirubinaemia is frequently underpinned by complex interactions of diverse biological and environmental risk factors across populations. It is our view therefore, that each LMIC still needs to develop context-specific guidelines for their own population [77]. Such an initiative should be broadened to involve key stakeholders including parent groups and community leaders. It may be helpful to prepare separate documents for each level of care to avoid information overload especially at the primary care level. While the scope of this framework is not exhaustive, like any of the existing guidelines, we believe that most of the recommendations will still be valuable to LMICs that did not meet our selection criteria. Finally, strong clinical and public health leadership at all levels will be required to surmount the challenges that typically mitigate against initiatives for improved child and newborn care in LMICs [78,79].

## Conclusions

In sharp contrast to the practice in most high-income nations, national guidelines for the effective management of severe hyperbilirubinaemia are rare in LMICs where the disease burden is greatest. In this paper, the authors have attempted to identify a number of key considerations for the effective management of hyperbilirubinaemia in LMICs that can be considered truly resource-poor, based on their HDI status. Most of the recommendations have been adapted from existing evidence or consensus-based guidelines in the industrialised world after extensive consultations at different stages with experts from various countries. Efforts were made to ensure that the proposed framework is consistent with universally accepted requirements for quality in healthcare. It is hoped that these recommendations will assist in the development of context-specific national guidelines and mobilisation of requisite resources for the care of infants with or at risk of severe hyperbilirubinaemia at all levels of healthcare delivery in LMICs.

## Additional files

Additional file 1: Table S1. Current published guidelines/recommendations for the management of hyperbilirubinaemia. Additional file 2: Table S2. Eligible low and middle-income countries (GNI per capita **≤**$6,000). Additional file 3: Figure S1. Guide to dermal staining with approximate serum bilirubin levels (Modified Kramer’s Scale). Additional file 4: Table S3. Bilirubin-Induced Neurological Dysfunction (BIND) II Scoring.

## Abbreviations

AAP: American Academy of Pediatrics
ABE: Acute Bilirubin Encephalopathy
BIND: Bilirubin induced neurologic dysfunction
CPT: Conventional phototherapy
CWG: Core Working Group
ET: Exchange transfusion
FS-PT: Filtered-Sunlight Phototherapy
G6PD: Glucose-6-Phosphate Dehydrogenase
GNI: Gross National Income
HDI: Human Development Index
LED: Light emitting diodes
LMIC: Low- or middle-income country
LMICs: Low and middle-income countries
NICE: National Institute for Health and Clinical Experience
NNJ: Neonatal Jaundice
PT: Phototherapy
TSB: Total Serum/Plasma Bilirubin
TcB: Transcutaneous Bilirubin
UNDP: United Nations Development Program
WHO: World Health Organization
