# Supplementary material for: Management of late-preterm and term infants with hyperbilirubinaemia in resource-constrained settings
Source: BMC Pediatr. 2015 Apr 12;15:39. doi: 10.1186/s12887-015-0358-z (PMC4409776; doi:10.1186/s12887-015-0358-z)

Table S1. Published guidelines/recommendations for the management of hyperbilirubinaemia

Country/region	Reference	Target population	Issuing body	AGREE II-GRS* Mean Rating (%)
Australia	1. Queensland Maternity and Neonatal Clinical Guidelines: Neonatal jaundice. Queensland Government, Australia, 2012. Available at: http://www.health.qld.gov.au/qcg/documents/g_jaundice.pdf	Preterm and full-term newborns	Regional Health Authority	89
Canada	2. Canadian Paediatric Society, Fetus and Newborn Committee. Guidelines for detection, management and prevention of hyperbilirubinemia in term and late preterm newborn infants (35 or more weeks' gestation). Paediatr Child Health 2007;12:1B–12B. Available at: www.cps.ca/english/statements/FN/fn07-02.htm.	Near and full-term infants (≥35weeks gestation)	Professional association	86
Europe/USA	3. Bhutani VK, Maisels MJ, Stark AR, Buonocore G; Expert Committee for Severe Neonatal Hyperbilirubinemia; European Society for Pediatric Research; American Academy of Pediatrics. Management of jaundice and prevention of severe neonatal hyperbilirubinemia in infants >or=35 weeks gestation. Neonatology.2008;94:63-7.	Near and full-term infants (≥35weeks gestation)	Professional association	63
Israel	4. Kaplan M, Merlob P, Regev R. Israel guidelines for the management of neonatal hyperbilirubinemia and prevention of kernicterus. J Perinatol. 2008;28:389-97.	Near and full-term infants (≥35weeks gestation)	Professional group	82
Italy	5. Romagnoli C, Barone G, Pratesi S, Raimondi F, Capasso L, Zecca E, Dani C; Task Force for Hyperbilirubinaemia of the Italian Society of Neonatology. Italian guidelines for management and treatment of hyperbilirubinaemia of newborn infants ≥ 35 weeks' gestational age. Ital J Pediatr. 2014 Jan 31;40(1):11.	Near and full-term infants (≥35weeks gestation)	Professional group	89
Netherlands	6. Dijk PH, de Vries TW, de Beer JJ; Dutch Pediatric Association. [Guideline 'Prevention, diagnosis and treatment of hyperbilirubinemia in the neonate with a gestational age of 35 or more weeks']. Ned Tijdschr Geneeskd. 2009;153:A93. [Translated into English]	Near and full-term infants (≥35weeks gestation)	Professional association	82
New Zealand	7. Auckland District Health Board Newborn Services Clinical Guideline: Management of Neonatal Jaundice, New Zealand, 2012. Available at: http://www.adhb.govt.nz/newborn/Guidelines/GI/Jaundice.htm	Preterm and full-term newborns	Regional Health Authority	55
Norway	8. Bratlid D, Nakstad B, Hansen TW. National guidelines for treatment of jaundice in the newborn. Acta Paediatr. 2011;100:499-505.	Preterm and full-term newborns	Professional association	74
Switzerland	9. Swiss Society of Neonatology. Assessment and Treatment of Jaundiced Newborn Infants 35 0/7 or more Weeks of Gestation. Elaborated by the working group consisting of: Arlettaz R, Blumberg A, Buetti L, Fahnenstich H, Mieth D, Roth-Kleiner M. Available at: http://www.neonet.ch/assets/pdf/2006_Bili-Empfehlungen_e_final.pdf	Near and full-term infants (≥35weeks gestation)	Professional association	76
UK	10. National Institute for Health and Clinical Excellence. Neonatal jaundice. (Clinical guideline 98.) 2010. Available at: www.nice.org.uk/CG98.	Preterm and full-term newborns	National Health Authority	99
USA	11. American Academy of Pediatrics Subcommittee on Hyperbilirubinemia. Management of hyperbilirubinemia in the newborn infant 35 or more weeks of gestation. Pediatrics. 2004;114:297-316;<br>• Ip S, Chung M, Kulig J, O'Brien R, Sege R, Glicken S, Maisels MJ, Lau J; American Academy of Pediatrics Subcommittee on Hyperbilirubinemia. An evidence-based review of important issues concerning neonatal hyperbilirubinemia. Pediatrics. 2004;114:e130-53.<br>• Maisels MJ, Bhutani VK, Bogen D, Newman TB, Stark AR, Watchko JF. Hyperbilirubinemia in the newborn infant >or=35 weeks' gestation: an update with clarifications. Pediatrics. 2009;124:1193-8	Near and full-term infants (≥35weeks gestation)	Professional association	94
	12. Academy of Breastfeeding Medicine Protocol Committee. ABM clinical protocol #22: guidelines for management of jaundice in the breastfeeding infant equal to or greater than 35 weeks' gestation. Breastfeed Med. 2010;5:87-93.	Near and full-term infants (≥35weeks gestation)	Professional association	88
All developing countries	13. World Health Organization. Pocket book of hospital care for children: Second edition. Guidelines for the management of common childhood illnesses. Geneva, Switzerland: WHO. 2013.	Preterm and full-term newborns	WHO	66

Ghana	14. Ministry of Health, Neonatal Jaundice: problems of the newborn in Standard Treatment Guidelines, Sixth edition. Republic of Ghana: 2010. Page 80. Available at: http://apps.who.int/medicinedocs/documents/s18015en/s18015en.pdf	Distinction not specified	National Health Authority	41
India	15. National Neonatal Forum, India. Management of neonatal hyperbilirubinemia. Available at: http://www.nnfpublication.org.	Preterm and full-term newborns	Professional group	88
	16. Mishra S, Agarwal R, Deorari AK, Paul VK. Jaundice in the newborns. Indian J Pediatr. 2008;75:157-63.	Preterm and full-term newborns	Professional group	77
	17. Postgraduate Institute of Medical Education and Research (PGIMER). Jaundice. The PGI NICU Handbook of Protocols Fourth Edition, Dutta s & Kumar P, Editors. Chandigarh, India. 2010.	Preterm and full-term newborns	Research Institute	81
Kenya	18. Ministry of Medical Services and Ministry of Public Health and Sanitation. Clinical management and referral guidelines – Vol II: Clinical guidelines for management and referral of common conditions at levels 2-3: Primary Care. Republic of Kenya, 2009. Pages 144-146. http://chs.uonbi.ac.ke/sites/default/files/chs/chs/Clinical%20Guidelines%20Vol%20II%20Final.pdf	Distinction not specified	National Health Authority	47
	19. Ministry of Health, Republic of Kenya. Basic paediatric protocols for ages up to 5 years. Nairobi, November 2013 Edition. pp 40-42.	Preterm and full-term newborns	National Health Authority	76
Malaysia	20. Ministry of Health Malaysia. Management of jaundice in healthy term newborns. Clinical Practice Guidelines. 2003. Available at: http://www.acadmed.org.my/index.cfm?&menuid=67	Near and full-term infants (≥35weeks gestation)	National Health Authority	83
South Africa	21. Horn AR, Kirsten GF, Kroon SM, Henning PA, Möller G, Pieper C, Adhikari M, Cooper P, Hoek B, Delport S, Nazo M, Mawela B. Phototherapy and exchange transfusion for neonatal hyperbilirubinaemia: neonatal academic hospitals' consensus guidelines for South African hospitals and primary care facilities. S Afr Med J. 2006;96:819-24.	Preterm and full-term newborns	Professional groups	83

Sources: Electronic search of Guideline Clearinghouses (www.guideline.gov and www.g-i-n.net/), PubMed, Scopus, Ovid EMBASE and Cumulative Index to Nursing and Allied Health Literature (CINAHL), reference list of guidelines and review papers, and suggestions from expert review panel.

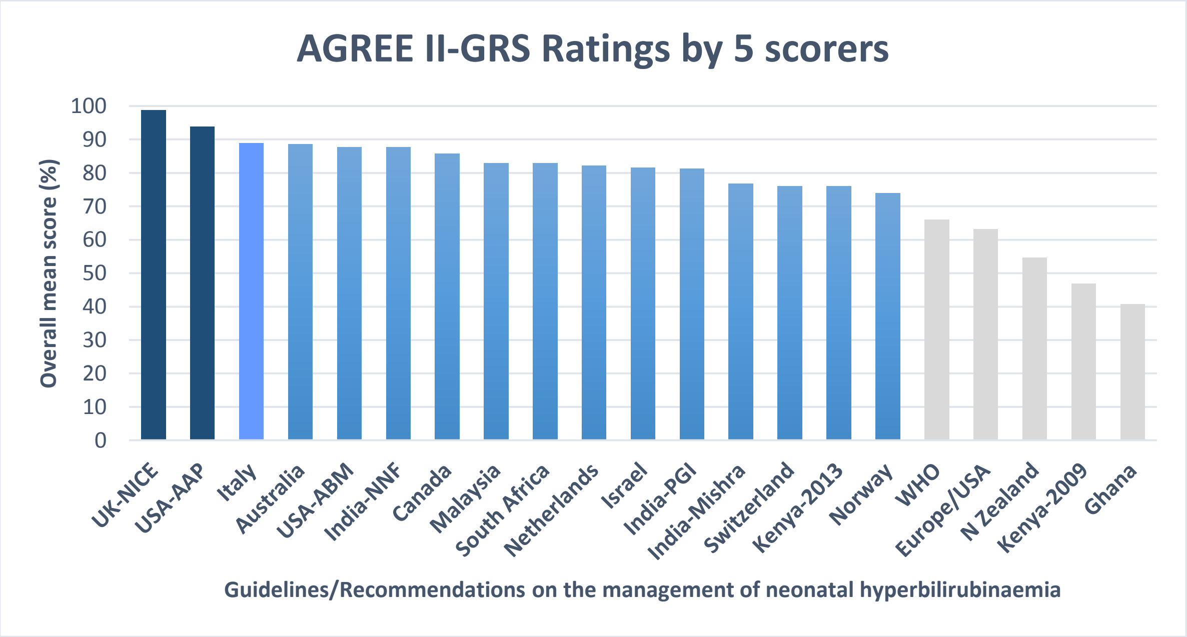

Supplement: Additional file 1: Table S1. — Current published guidelines/recommendations for the management of hyperbilirubinaemia. [file 12887_2015_358_MOESM1_ESM.pdf]
