# Supplementary material for: Management of late-preterm and term infants with hyperbilirubinaemia in resource-constrained settings
Source: BMC Pediatr. 2015 Apr 12;15:39. doi: 10.1186/s12887-015-0358-z (PMC4409776; doi:10.1186/s12887-015-0358-z)
Supplement: Additional file 2: Table S2. — Eligible low and middle-income countries (GNI per capita ≤$6,000). [file 12887_2015_358_MOESM2_ESM.pdf]

Table S2. Eligible low and middle-income countries (GNI per capita ≤$6,000)

SN	COUNTRY	Region	HDI RANK 2012	HDI	Life Expectancy (Years)	GNI per capita ($)	Annual Livebirths ('000)	Hospital Delivery (%)
1	**Afghanistan**	SOA	175	0.374	49.1	1,000	1,408	33
2	**Angola**	SSA	148	0.508	51.5	4,812	803	46
3	**Armenia**	ECA	87	0.729	74.4	5,540	47	99
4	**Bangladesh**	SOA	146	0.515	69.2	1,785	3,016	29
5	**Belize**	LAC	96	0.702	76.3	5,327	8	89
6	**Benin**	SSA	166	0.436	56.5	1,439	356	87
7	**Bhutan**	SOA	140	0.538	67.6	5,246	15	63
8	**Bolivia, Plurinational State of**	LAC	108	0.675	66.9	4,444	264	68
9	**Burkina Faso**	SSA	183	0.343	55.9	1,202	730	66
10	**Burundi**	SSA	178	0.355	50.9	544	288	60
11	**Cambodia**	EAP	138	0.543	63.6	2,095	317	54
12	**Cameroon**	SSA	150	0.495	52.1	2,114	716	61
13	**Cape Verde**	SSA	132	0.586	74.3	3,609	10	76
14	**Central African Republic**	SSA	180	0.352	49.1	722	156	53
15	**Chad**	SSA	184	0.34	49.9	1,258	511	16
16	**Comoros**	SSA	169	0.429	61.5	986	28	
17	**Congo**	SSA	142	0.534	57.8	2,934	145	92
18	**Congo, Democratic Republic of the**	SSA	186	0.304	48.7	319	2,912	75
19	**Côte d'Ivoire**	SSA	168	0.432	56	1,593	679	57
20	**Cuba**	LAC	59	0.78	79.3	5,539	110	100
21	**Djibouti**	MEN	164	0.445	58.3	2,350	26	87
22	**Egypt**	MEN	112	0.662	73.5	5,401	1,886	72
23	**El Salvador**	LAC	107	0.68	72.4	5,915	126	85
24	**Eritrea**	SSA	181	0.351	62	531	193	26
25	**Ethiopia**	SSA	173	0.396	59.7	1,017	2,613	10
26	**Fiji**	EAP	96	0.702	69.4	4,087	18	
27	**Gambia**	SSA	165	0.439	58.8	1,731	67	56
28	**Georgia**	ECA	72	0.745	73.9	5,005	51	98
29	**Ghana**	SSA	135	0.558	64.6	1,684	776	67
30	**Guatemala**	LAC	133	0.581	71.4	4,235	473	51
31	**Guinea**	SSA	178	0.355	54.5	941	394	39
32	**Guinea-Bissau**	SSA	176	0.364	48.6	1,042	59	42
33	**Guyana**	LAC	118	0.636	70.2	3,387	13	89
34	**Haiti**	LAC	161	0.456	62.4	1,070	266	25
35	**Honduras**	LAC	120	0.632	73.4	3,426	205	67
36	**India**	SOA	136	0.554	65.8	3,285	27,098	47
37	**Indonesia**	EAP	121	0.629	69.8	4,154	4,331	55
38	**Iraq**	MEN	131	0.59	69.6	3,557	1,144	65
39	**Jordan**	MEN	100	0.7	73.5	5,272	154	99
40	**Kenya**	SSA	145	0.519	57.7	1,541	1,560	43
41	**Kiribati**	EAP	121	0.629	68.4	3,079	22	66
42	**Kyrgyzstan**	ECA	125	0.622	68	2,009	131	97
43	**Lao People's Democratic Republic**	EAP	138	0.543	67.8	2,435	140	17
44	**Lesotho**	SSA	158	0.461	48.7	1,879	60	59
45	**Liberia**	SSA	174	0.388	57.3	480	157	37
46	**Madagascar**	SSA	151	0.483	66.9	828	747	35
47	**Malawi**	SSA	170	0.418	54.8	774	686	73
48	**Mali**	SSA	182	0.344	51.9	853	728	45
49	**Marshall Islands**	EAP		..	72.3	4,040	27	85
50	**Mauritania**	SSA	155	0.467	58.9	2,174	118	48
51	**Micronesia, Federated States of**	EAP	117	0.645	69.2	3,352	3	
52	**Moldova, Republic of**	ECA	113	0.66	69.6	3,319	44	99
53	**Mongolia**	EAP	108	0.675	68.8	4,245	65	99
54	**Morocco**	MEN	130	0.591	72.4	4,384	620	73
55	**Mozambique**	SSA	185	0.327	50.7	906	889	58
56	**Myanmar**	EAP	149	0.498	65.7	1,817	824	36
57	**Namibia**	SSA	128	0.608	62.6	5,973	60	81
58	**Nepal**	SOA	157	0.463	69.1	1,137	722	35
59	**Nicaragua**	LAC	129	0.599	74.3	2,551	138	74
60	**Niger**	SSA	186	0.304	55.1	701	777	17
61	**Nigeria**	SSA	153	0.471	52.3	2,102	6,458	35
62	**Pakistan**	SOA	146	0.515	65.7	2,566	4,764	41
63	**Palestine, State of**	MEN	110	0.67	73	3,359	33	
64	**Papua New Guinea**	EAP	156	0.466	63.1	2,386	208	52
65	**Paraguay**	LAC	111	0.669	72.7	4,497	158	82
66	**Philippines**	EAP	114	0.654	69	3,752	2,358	44
67	**Rwanda**	SSA	167	0.434	55.7	1,147	449	69
68	**Samoa**	EAP	96	0.702	72.7	3,928	4	81
69	**Sao Tome and Principe**	SSA	144	0.525	64.9	1,864	5	79
70	**Senegal**	SSA	154	0.47	59.6	1,653	471	73
71	**Sierra Leone**	SSA	177	0.359	48.1	881	227	50
72	**Solomon Islands**	EAP	143	0.53	68.2	2,172	17	85
73	**Somalia**	SSA		..	51.5	150	416	9
74	**South Sudan**	SSA		..	..	..		
75	**Sri Lanka**	SOA	92	0.715	75.1	5,170	373	98
76	**Sudan**	SSA	171	0.414	61.8	1,848	1,447	21
77	**Swaziland**	SSA	141	0.536	48.9	5,104	35	80
78	**Syrian Arab Republic**	MEN	116	0.648	76	4,674	466	78
79	**Tajikistan**	ECA	125	0.622	67.8	2,119	194	88
80	**Tanzania, United Republic of**	SSA	152	0.476	58.9	1,383	1,913	50
81	**Timor-Leste**	EAP	134	0.576	62.9	5,446	44	22
82	**Togo**	SSA	158	0.459	57.5	928	195	67
83	**Tonga**	EAP	95	0.71	72.5	4,153	3	98
84	**Tuvalu**	EAP		..	67.5	5,650		93
85	**Uganda**	SSA	161	0.456	54.5	1,168	1,545	57
86	**Uzbekistan**	ECA	114	0.654	68.6	3,201	589	97
87	**Vanuatu**	EAP	124	0.626	71.3	3,960	7	80
88	**Vietnam**	EAP	127	0.617	75.4	2,970	1,458	92
89	**Yemen**	MEN	160	0.458	65.9	1,820	940	24
90	**Zambia**	SSA	163	0.448	49.4	1,358	622	48
91	**Zimbabwe**	SSA	172	0.397	52.7	424	377	65

HDI: Human Development Index; GNI: Gross National Income; World Regions: East Asia & Pacific (EAP), Europe & Central Asia (ECA), Latin America & Caribbean (LAC), Middle East & North Africa (MEN), South of Asia (SOA) and Sub-Saharan Africa (SSA).

Notes: The human development index (HDI), is published by the United Nations Development Program (UNDP). It is a robust composite measure of the average achievement in three basic dimensions of human development namely: a long and healthy life (health), knowledge (education) and a decent standard of living (income). By world regions, 42 (46%) countries are from Sub-Saharan Africa, 18 (20%) from East Asia & Pacific, 10 (11%) from Latin America & Caribbean, 8 (9%) from Middle East & North Africa, 7 (8%) from South Asia and 6 (6%) from Europe & Central Asia. About a third (33 countries) have at least half a million live-births annually, account for over half (58%) of global births and have a median institutional delivery rate of 47% (IQR:35 -65%).
