# Supplementary material for: Management of late-preterm and term infants with hyperbilirubinaemia in resource-constrained settings
Source: BMC Pediatr. 2015 Apr 12;15:39. doi: 10.1186/s12887-015-0358-z (PMC4409776; doi:10.1186/s12887-015-0358-z)
Supplement: Additional file 3: Figure S1. — Guide to dermal staining with approximate serum bilirubin levels (Modified Kramer’s Scale). [file 12887_2015_358_MOESM3_ESM.pdf]

Zone	Baby’s body area	Approximate bilirubin level	Diagram
**1**	Head and neck	6 mg/dl<br>(100 µmol/L)	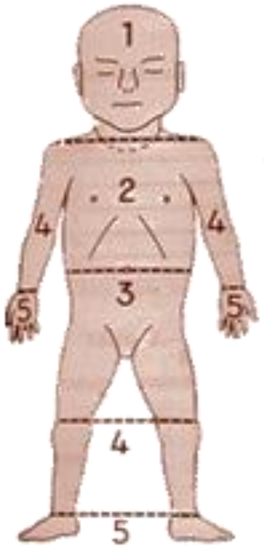
**2**	Upper body (chest)	9 mg/dl<br>(150 µmol/L)	
**3**	Lower body, below the belly button and upper thighs and arms	12 mg/dl<br>(200 µmol/L)	
**4**	Lower legs and forearms	15 mg/dl<br>(250 µmol/L)	
**5**	Hands and feet	>15 mg/dl<br>(>250 µmol/L)	

[Manzar S. (1999) Cephalo-caudal progression of jaundice: a reliable, non-invasive clinical method to assess the degree of neonatal hyperbilirubinaemia. J Trop Pediatr 45:312–313]
