# Supplementary material for: Management of late-preterm and term infants with hyperbilirubinaemia in resource-constrained settings
Source: BMC Pediatr. 2015 Apr 12;15:39. doi: 10.1186/s12887-015-0358-z (PMC4409776; doi:10.1186/s12887-015-0358-z)
Supplement: Additional file 4: Table S3. — Bilirubin-Induced Neurological Dysfunction (BIND) II Scoring. [file 12887_2015_358_MOESM4_ESM.pdf]

Table S3. Bilirubin-Induced Neurological Dysfunction (BIND) II SCORING		
**CLINICAL SIGN (score most severe sign)**	**SCORE**	**SEVERITY**
**MENTAL STATUS**		
0 Normal	0	None
0 Sleepy but arousable<br>0 Decreased feeding	1	Mild
0 Lethargy<br>0 Poor suck and/or<br>0 Irritable/jittery with short-term strong suck	2	Moderate
0 Semi-coma<br>0 Apnea<br>0 Seizures<br>0 Coma	3	Severe
**MUSCLE TONE**		
0 Normal	0	None
0 Persistent mild hypotonia	1	Mild
0 Moderate hypotonia<br>0 Moderate hypertonia<br>0 Increasing arching of neck and trunk on stimulation without spasms of arms and legs and without trismus	2	Moderate
0 Persistent retrocolis<br>0 Opisthotonos<br>0 Crossing or scissoring of arms or legs but without spasms of arms and legs and without trismus	3	Severe
**CRY PATTERN**		
0 Normal	0	None
0 High-pitched	1	Mild
0 Shrill	2	Moderate
0 Inconsolable crying or<br>0 Cry weak or absent in child with previous history of high pitched or shrill cry	3	Severe
**OCCULOMOTOR / EYE MOVEMENTS / FACIES**		
0 Normal	0	None, Mild
0 Sun-setting<br>0 Paralysis of upward gaze<br>0 Disconjugate eye movements<br>0 Blank stare<br>0 Aimless eye movements	3	Severe
**TOTAL BIND II (ABE SCORE)**		

Notes: Scores of 1-4 are consistent with **mild ABE** but cannot be differentiated from sepsis or other neonatal illnesses without ancillary testing, such as ABR and/or MRI.

Scores 5 to 8 are consistent with **moderate ABE**

Scores ≥ 9 are consistent with **severe ABE**

The BIND score may include different stages for different categories, for example scores of 4 or 5 might represent the sum of one for cry and two for muscle tone and/or arousal state.

Adapted from:

1. Johnson L, Bhutaini VK, Karp K, Sivieri EM, Shapiro SM. (2009) Clinical report from the pilot USA Kernicterus Registry (1992 to 2004). *J Perinatol* 29; Suppl 1:S25-45.

2. Slusher T, Olusanya B. (2012) Neonatal Jaundice in Low-Middle Income Countries: In: Stevenson D, Maisels M, Watchko J, editors. Neonatal Jaundice Transient Unconjugated Hyperbilirubinemia of the Newborn. New York. McGraw Hill.
